# Editorial: Machine learning and artificial intelligence for smart agriculture, volume II

**DOI:** 10.3389/fpls.2023.1166209

**Published:** 2023-04-20

**Authors:** Shanwen Zhang, Chuanlei Zhang, Dong Sun Park, Sook Yoon

**Affiliations:** ^1^ Information Engineering College, Xijing University, Xi’an, China; ^2^ College of Artificial Intelligence, Tianjin University of Science and Technology, Tianjin, China; ^3^ Department of Electronics Engineering, Jeonbuk National University, Jeonju, Republic of Korea; ^4^ Department of Computer Engineering, Mokpo National University, Muan, Republic of Korea

**Keywords:** AUAV, crop type mapping, crop phenotypic analysis, crop disease-pest control, artificial intelligence

## 1 Introduction

Currently, AI is being widely used in various agricultural scenarios, including intelligent perception, real-time field monitoring, intelligent early warning, disease and pest detection, and intelligent decision-making for crop production environments. With the help of AI, farmers can now detect whether there are any diseases and pests, whether they need to use pesticides, and whether their plant protection practices are effective. This special edition focuses on several issues that still require further study and discussion, such as agricultural unmanned aerial vehicles, crop type mapping, crop phenotypic analysis, and the identification of crop diseases and pests in sustainable and intelligent phytoprotection.

## 2 Agricultural unmanned aerial vehicle

Agricultural unmanned aerial vehicles (AUAVs) integrate robots, AI, big data, and the Internet of Things. They have been widely applied to various agricultural operations, such as seed sowing, land monitoring, crop disease and pest detection, and pesticide and fertilizer spraying. AUAVs greatly improve agricultural production efficiency and liberate the labor force (Kim et al., 2019). They are becoming a new force in the field of precision agricultural aviation (Wang et al., 2019). Compared to traditional agricultural machinery, they are small, lightweight, and easy to transport, and have flexible flight control. AUAVs are characterized by precision operation, high efficiency, environmental friendliness, intelligence, and ease of use. However, in many cases, real-time changes in the AUAV load during flight can affect its speed, accuracy, and flight path stability. Xu et al. (Wei et al.) proposed a flight dynamics model for achieving AUAV flight trajectory stability using a PID controller and robust T-S fuzzy control method. This model can achieve certain stability in the flight path against load perturbations for different mission requirements. With crop growth data recorded by AUAVs, farmers can analyze their crops and make informative decisions based on accurate crop growth information.

## 3 Crop type mapping

Large-scale and accurate CTM plays a critical role in agricultural management, including field-scale crop monitoring, optimizing crop distribution, and achieving agricultural intensification for sustainable development and food security. However, it is challenging due to factors such as crop diversity, inter-class spectral similarity, and intra-class variability. Traditional CTM methods rely on remote sensing images (RSIs) as data sources, but cloud cover and limited availability of optical images during critical crop growth periods can impede the accuracy of RSIs, particularly in hot and rainy areas (Yang et al., 2019). Moreover, the irregular time series and limited coverage of remote sensing data further complicate CTM. To overcome these challenges, recent studies have proposed deep learning-based CTM methods that outperform traditional machine learning methods, leveraging advancements in Earth observation satellites and deep learning technology (Pott et al., 2021). For instance, in Bian et al. designed a channel attention U-Net model that integrates shallow CNN, U-Net, and channel attention mechanism to improve the spectral feature extraction ability. This approach can better handle the problem of inconsistent availability of remote sensing data due to cloud and rainy weather. Future research should continue to focus on addressing this problem to realize large-scale CTM for precision agriculture management and macro-control of food production.

## 4 Crop phenotypic analysis

Overall, crop phenotypic analysis (CPA) is an essential tool in understanding the various factors affecting crop growth and providing timely data for crop managers. Traditional CPA methods rely on manual operations, which are time-consuming and labor-intensive, and the analysis results may be unstable and inaccurate (Song et al., 2021). To overcome these challenges, machine vision and deep learning techniques can be used to achieve rapid and accurate analysis of crop phenotypic characteristics (Xiong et al., 2021). In Zhang et al. proposed a three-stage multi-branch self-correcting trait estimation network (TMSCNet) for CPA, which can provide a scientific basis for real-time monitoring of crop growth. Additionally, seed morphology analysis is important for understanding the taxonomic relationship of various plant families and genera and for developing higher-yield and better-quality crop varieties. In Seki et al. used image-based phenotyping to develop a quantitative method for measuring seed morphology traits, even for small crop seed sizes, through deep learning. This approach can accelerate the discovery of the genetic basis of small morphological characteristics, such as seed size and shape.

## 5 Crop disease-pest control

Crop disease and pest identification is a critical aspect of agriculture that can help reduce pesticide use and increase agricultural productivity in a sustainable manner. Traditional methods of identification such as support vector machines, Naive Bayes and BP neural networks are not suitable for large area disease-pest identification in the field due to low recognition rate and weak generalization. In contrast, deep learning methods based on convolutional neural networks (CNN) have shown remarkable results and have strong generalization (Gu et al., 2021). Pre-trained VGG and ResNet 50 architectures based on the ImageNet dataset are commonly used due to the scarcity of images of crop disease-pests. To improve the identification accuracy of small insect targets, S-ResNet has been constructed based on ResNet, which has a 7% improvement in identification accuracy in Wang et al.. Deep learning methods require powerful computing power and large training datasets, which make them difficult to deploy on mobile devices (Chen et al., 2021). Future research efforts should focus on developing lightweight Siamese networks and incorporating other data forms such as geographic location, disease-pest incidence history, and weather trends to enhance the accuracy and reliability of disease-pest recognition systems.

## Author contributions

CZ worked on the editing of the papers on Crop Type Mapping, Crop Phenotypic Analysis and Crop Disease-pest Control. SZ worked on the editing of AUAV related papers. DP worked on the editing of AUAV related papers. SY worked on the review of the editorial. All authors contributed to the article and approved the submitted version.
